# Gut microbiota and brain aging: a comparative review of African and western populations

**DOI:** 10.3389/fnagi.2026.1740408

**Published:** 2026-02-12

**Authors:** Beulah Favour Ortutu, Abidemi Oluwasanmi Okin, Kwame Osei Darkwah, Uju Maryanne Onuorah, Abdulkadir Yusif Maigoro, Gideon Onyedikachi Iheme

**Affiliations:** 1 Department of Nutrition, Texas A&M University, College Station, TX, United States; 2 Department of Human Nutrition and Dietetics, Michael Okpara University of Agriculture, Umudike, Nigeria; 3 Department of Family and Consumer Sciences, North Carolina Agricultural and Technical State University, Greensboro, NC, United States; 4 Department of Cell and Molecular Biology, School of Science and Engineering, Tulane University, New Orleans, LA, United States; 5 Department of International Public Health, Liverpool School of Tropical Medicine, Liverpool, United Kingdom; 6 Department of Pharmacy and Health and Nutrition Sciences, University of Calabria, Rende, Italy; 7 Department of Nutrition and Dietetics, Faculty of Agricultural Sciences, University of Nigeria, Nsukka, Nigeria; 8 Convergence Research Center for Insect Vectors, Incheon, Republic of Korea; 9 Department of Food Studies, Nutrition and Dietetics, Uppsala University, Uppsala, Sweden

**Keywords:** Africa, brain aging, diet, gut microbiota, gut-brain axis, neurodegenerative disease

## Abstract

As the population ages, cognitive decline and neurodegenerative diseases have become major public health concerns. The human gut microbiota plays a major role in regulating neurodevelopment, neuroinflammation, and cognitive decline through the gut-brain axis. Emerging evidence reveals a possible association between alterations in gut microbial diversity and age-related neurological disorders, including Alzheimer’s disease and neurodegeneration. Regional and dietary differences shape the gut microbiome. These variations may, in turn, be associated with differences in brain aging across populations. Several cross-sectional studies indicate that rural African communities consuming predominantly fiber-rich diets exhibit distinct gut microbiota profiles characterized by increased abundance of genera, including *Prevotella*, *Faecalibacterium*, and *Ruminococcus*. These microbial configurations have been associated with improved gut barrier integrity, reduced systemic inflammation, and enhanced production of short-chain fatty acids in some preclinical and human studies. All these factors have been studied as potential mechanisms linked to delayed brain aging. Furthermore, epidemiological reports suggest lower prevalence rates of dementia and other neurodegenerative disorders in these populations, although such comparisons may be influenced by differences in study design, diagnosis, and case ascertainment across regions. This narrative review synthesized current understanding of the gut microbiota’s role in brain aging, summarized available data on gut microbiota composition in African versus Western populations, and explored the pathways by which traditional African diets may contribute to neuroprotection. By critically examining this evidence and highlighting major research gaps, the review advocates for region-specific investigations and future longitudinal studies to validate causal links.

## 1 Introduction

Aging is characterized by a progressive decline in physical, physiological, and cognitive functions, increasing susceptibility to chronic diseases, and mortality (Dharmarajan et al., 2012; Guo et al., 2022). Brain aging encompasses physical and biological changes that occur over time, while age-related cognitive decline reflects the gradual deterioration of learning, memory, and reasoning abilities (Thakur et al., 2016). In the brain, aging involves synaptic loss, reduced plasticity, and cumulative cellular damage from oxidative stress, which are processes commonly observed in neurodegenerative conditions such as Alzheimer’s disease (AD), Parkinson’s disease (PD), and dementia (Tchekalarova and Tzoneva, 2023; Zia et al., 2021). The prevalence of these neurodegenerative diseases has been shown to increase with age. AD affects approximately one in ten older adults (Alzheimer’s and Dementia, 2024), while PD prevalence triples among individuals over 55 years (Peng et al., 2025).

With global populations aging rapidly, the burden of neurodegenerative diseases (NDs) has become a critical public health and economic challenge. In sub-Saharan Africa, dementia prevalence ranges from 2% to 20% among older adults, with estimates reaching 18% in some communities (Akinyemi et al., 2022). However, these estimates vary widely and may reflect differences in diagnostic criteria, study methodology and healthcare access (Akinyemi et al., 2022; Farrell et al., 2024; Kamoga et al., 2019). Projections suggest that tens of millions of Africans may be at risk of PD in the coming decades as a result of urbanization (Onohuean et al., 2022; Pereira et al., 2024). Rapid urbanization threatens beneficial microbiota growth and may be associated with increasing neurodegenerative disease risk (Huang et al., 2023; Zhang H. et al., 2022). In Western societies, more than 150 million people are currently affected by NDs, a number expected to exceed 152 million by 2060, with PD cases projected to reach 25 million by 2050 (Onohuean et al., 2022; Peng et al., 2025).

The gut-brain microbiome (GBM) axis is a bidirectional communication network between the gut microbiota and the central nervous system. Increasing evidence highlights that the GBM axis plays a key role in regulating neurodevelopment, neuroinflammation, and cognitive aging (Loh et al., 2024). Dysbiosis of the gut microbiota has been implicated in promoting depressive-like behaviors and cognitive decline, both in animal models and human studies (Chu et al., 2019; Hashim and Makpol, 2022). Diet plays a pivotal role in shaping the gut microbiota. Dietary fiber supports microbial diversity and drives the production of short-chain fatty acids (SCFAs), which have been hypothesized to exert neuroprotective and anti-inflammatory effects (Berding et al., 2021). However, most microbiome-brain aging studies have focused on Western populations, leaving a significant gap in our understanding of African populations, who commonly consume fiber-rich diets known to foster beneficial microbiota growth. Given the distinctive dietary patterns in many African communities, exploring their potential role in modulating gut-brain interactions and neurodegenerative disease risk is necessary.

To bridge this gap, this narrative review synthesizes current knowledge on the gut microbiota’s role in brain aging, compares microbial composition across African and Western populations, and examines how traditional African dietary patterns may support neuroprotection through the gut-brain axis. By highlighting key evidence and research gaps, this work aims to inform future region-specific investigations and global strategies for promoting healthy brain aging.

## 2 Methods

This narrative review literature search was conducted across databases, including PubMed, Scopus, Web of Science, and Google Scholar. These databases were selected to ensure we covered most of the literature in the field. Keywords such as “gut microbiota,” “brain aging,” “traditional African diets,” “rural African diet,” “African diet,” “gut microbiome composition in Africa,” “neurodegeneration,” “microbiota-gut-brain axis,” “Africa,” “Western diet,” and “dietary fiber” with the Boolean operators (AND, OR) used in various combinations. Relevant studies published in English between 2000 and 2025 were included. Additional references were identified through manual screening of bibliographies and review articles. Peer-reviewed original research articles, reviews, and meta-analyses published in English were included. African studies focusing on microbial signatures in diseased conditions were included only when they reported data from healthy control groups. Priority was given to human studies examining the gut-brain axis and dietary influences on microbiota composition, particularly those involving African or Western populations. Mechanistic animal or cellular studies were included when they provided critical insights into pathways relevant to neuroinflammation, SCFA production, and brain aging. While a formal quality assessment was not performed due to the narrative nature of this review, studies were prioritized and selected based on explicit reporting of key methodological details, including adequate sample characteristics, clear data collection procedures, and appropriate analytical methods, to ensure a sound foundation for synthesis.

Although this review follows a narrative framework, additional steps were taken to enhance transparency and rigor (Supplementary Figure 1). An initial screening of 201 articles was performed based on title and abstract relevance, followed by full-text assessment of studies most directly addressing gut microbiota composition, dietary patterns, and brain aging or neurodegeneration. Greater weight was given to human observational and interventional studies conducted in African and Western populations, while animal studies were used primarily to support mechanistic pathways linking microbial metabolites to neuroinflammation, mitochondrial function, and cognitive aging. African and Western studies were intentionally synthesized in parallel to allow comparative interpretation rather than direct quantitative comparison. Microbiome profiling studies using 16S rRNA sequencing constituted the majority of the human literature and were therefore most frequently cited, while shotgun metagenomic studies were incorporated when available to support functional inferences. This approach reflects the current distribution of evidence in the field and is consistent with the exploratory aims of a narrative review.

## 3 The role of gut microbiota in brain aging

**FIGURE 1:**
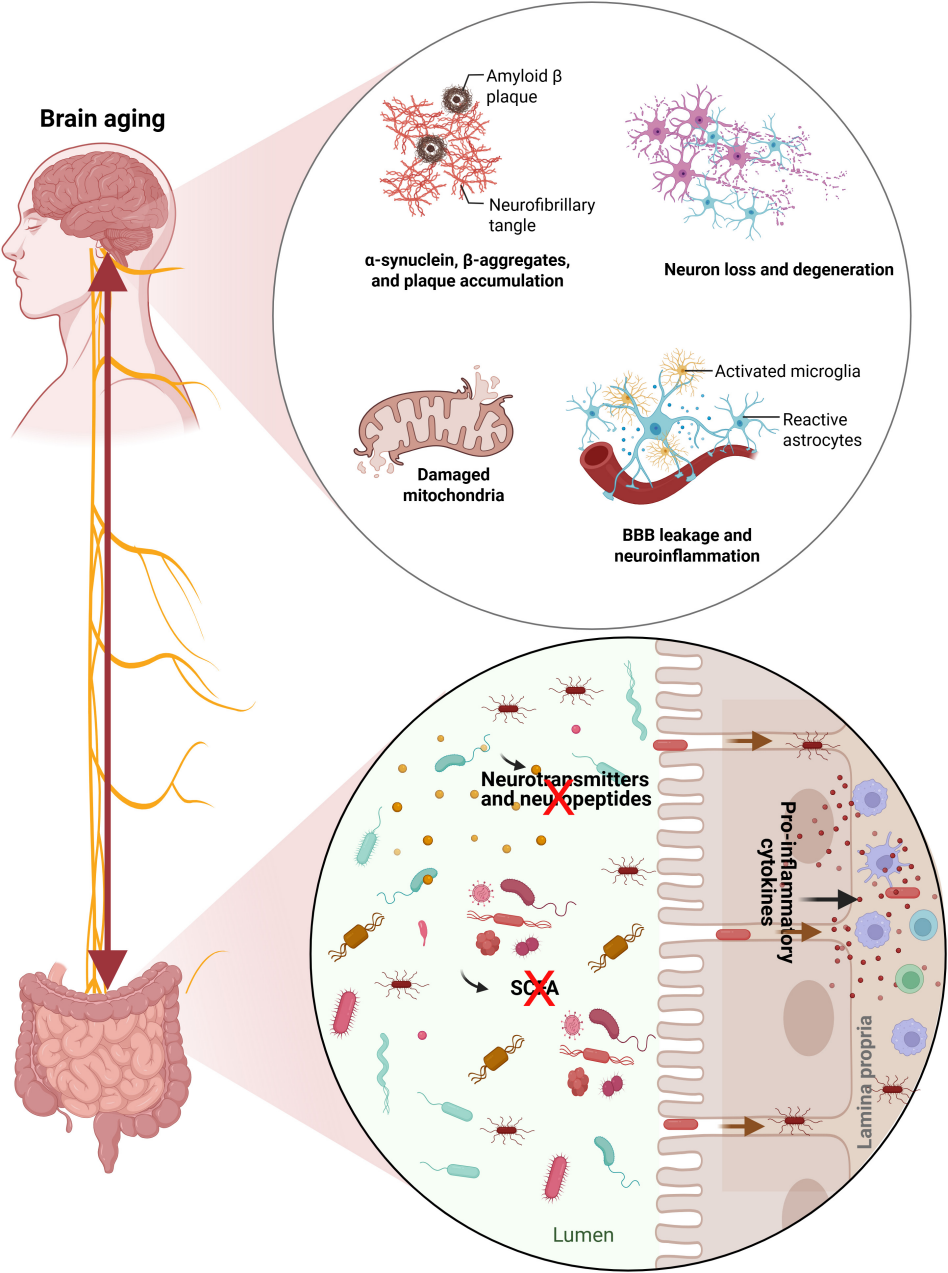
Gut-brain axis mechanisms linking gut microbiota alterations to brain aging. Alteration in the gut environment is characterized by reduced microbial diversity, an increased abundance of pathogenic bacteria, and a loss of beneficial SCFA-producing taxa. These changes lead to impaired gut barrier integrity, allowing microbial products and pro-inflammatory cytokines to translocate across the intestinal epithelium into the lamina propria and systemic circulation. In addition, reduced microbial synthesis of short-chain fatty acids (SCFAs), neurotransmitters, and neuroactive peptides disrupts immune regulation and neuroendocrine signaling. Signals originating from the altered gut microbiota reach the brain through multiple pathways, including circulating inflammatory mediators, microbial metabolites, and neural signaling through the vagus nerve. This reflects the bidirectional nature of gut-brain communication. In the aging brain, these processes are associated with blood-brain barrier (BBB) dysfunction, neuroinflammation, microglial activation, reactive astrogliosis, mitochondrial damage, and progressive neuronal loss. Accumulation of misfolded proteins, including α-synuclein and amyloid-β plaques, further contributes to synaptic dysfunction and cognitive decline (Carloni and Rescigno, 2023; Jing et al., 2024; Li et al., 2021). Collectively, the figure illustrates how gut microbiota alterations may drive neuroinflammatory and neurodegenerative processes, while brain aging itself can feedback to further disrupt gut microbial composition, reinforcing a pathogenic gut-brain loop. This figure was created with Biorender.Com. SCFAs, short-chain fatty acids.

Age-related alterations in gut microbiota composition contribute to a chronic low-grade inflammatory state, termed “inflammaging” (Fransen et al., 2017; Gyriki et al., 2025), which plays a central role in brain aging (Alsegiani and Shah, 2022). With advancing age, gut microbial diversity declines, leading to compromised intestinal barrier integrity and increased systemic inflammation. These systemic changes can disrupt the blood-brain barrier (BBB), promote microglial activation, and accelerate cognitive decline (Jurcau et al., 2024; Loh et al., 2024). The gut microbiota plays a central role in the pathogenesis of NDs through the gut-brain axis, influencing neuroinflammation, immune responses, and metabolic homeostasis (Zhang H. et al., 2022). While data directly linking gut microbiota to normal brain aging remains limited, comprehensive reviews on the gut microbiota and healthy aging provide foundational insights and discuss broad associations with healthy aging (Donati Zeppa et al., 2022; Jing et al., 2024; Kim and Jazwinski, 2018). Neurodegenerative diseases such as Alzheimer’s and Parkinson’s diseases represent conditions of accelerated or pathological brain aging, sharing common mechanisms such as neuroinflammation, blood-brain barrier dysfunction, and microbiota-driven metabolic disturbances. Characterizing microbial signatures in these diseases provides a useful framework for understanding how age-associated microbial alterations may predispose individuals to, or reflect, brain aging processes. Figure 1 summarizes the mechanisms through which alterations in the gut environment contribute to brain aging.

### 3.1 Microbiota signatures in neurodegenerative diseases as models of accelerated brain aging

Recent studies reveal both shared and disease-specific gut microbial alterations across Parkinson’s disease (PD) and Alzheimer’s disease (AD), suggesting common pathways that may be influenced by microbiota-based interventions (Table 1). Verrucomicrobia consistently emerges as a recurrent signature, with increased abundance reported in PD and AD (Heravi et al., 2023; Koutsokostas et al., 2024; Nishiwaki et al., 2022; Rob et al., 2025). This increase is primarily driven by its genus, *Akkermansia*, which has been linked to gut barrier modulation and pro-inflammatory signaling, lipopolysaccharide (LPS) production and modulation of immune signaling pathways, including upregulation of CASP1, TRAF5, and STAT5B (Derrien et al., 2011; Zhang and Wang, 2023) While *Akkermansia* is generally recognized for its beneficial effects on gut health and barrier modulation in non-disease states (Chiantera et al., 2024; Gao et al., 2025), its sustained elevation specifically in neurodegenerative contexts has been associated with barrier dysfunction, systemic inflammation, and reduced levels of metabolites, suggesting a shift in its functional implications during disease progression (Jian et al., 2023).

**TABLE 1:** Microbiota signatures in neurodegenerative diseases.

	PD	AD	References
**Phylum**			
*Verrrucomicrobia*	**↑**	**↑**	Rob et al., 2025; Heravi et al., 2023
*Firmicutes*	**↓**	**↓**	Intili et al., 2023; Sun et al., 2019
*Bacteroidota*	**↑**	**↑**	Intili et al., 2023
*Actinobacteria*	**↑**	**↑**	Koutsokostas et al., 2024
*Proteobacteria*	**↑**	**↑**	Koutsokostas et al., 2024
**Family**			
*Akkermansiaceae*	**↑**	**↑**	Rob et al., 2025
*Lactobacillaceae*	**↑**	**↑**	Romano et al., 2021; Wu et al., 2023
*Bifidobacteriaceae*	**↑**	**↓**	Romano et al., 2021; Vogt et al., 2017
*Enterobacteriaceae*	**↑**	**↑**	Guo et al., 2025
*Prevotellaceae*	**↓**	**↓**	Chui et al., 2024; Heravi et al., 2023; Lwere et al., 2025
*Faecalibacteriaceae*	**↓**	**↓**	Heravi et al., 2023; Koutsokostas et al., 2024
*Ruminococcaceae*	**↓**	**↓**	Cilia et al., 2021; Koutsokostas et al., 2024
*Clostridiaceae*	**↑**	**↓**	Bullich et al., 2019; Hung et al., 2022; Koutsokostas et al., 2024
*Veillonellaceae*	**–**	**↓**	Liang et al., 2024
*Methanobacteriaceae*	**↑**	**–**	Wallen et al., 2022
*Megasphaeraceae*	**↑**	**–**	Wallen et al., 2022
*Lachnospiraceae*	**↓**	**↓**	Li et al., 2017
*Peptostreptococcaceae*	**↓**	**↓**	Varesi et al., 2022; Vogt et al., 2017
**Genus**			
*Akkermansia*	**↑**	**↑**	Heravi et al., 2023; Rob et al., 2025
*Bifidobacterium*	**↑**	**↓**	Rasoulian et al., 2025; Romano et al., 2021; Vogt et al., 2017
*Lactobacillus*	**↑**	**↑**	Chui et al., 2024; Romano et al., 2021
*Faecalibacterium*	**↓**	**–**	Heravi et al., 2023; Koutsokostas et al., 2024; Liu et al., 2020
*Blautia*	**↓**	**–**	Rob et al., 2025
*Ruminococcus*	**↓**	**–**	Li et al., 2017
*Roseburia*	**↓**	**–**	Wallen et al., 2022
*Bacteroides*	**–**	**↑**	Rob et al., 2025
*Alistipes*	**–**	**↑**	Li et al., 2024
*Prevotella*	**↓**	**↓**	Chui et al., 2024; Heravi et al., 2023
*Escherichia*	**–**	**–**	
*Megasphaera*	**↑**	**–**	Wallen et al., 2022
*Methanobrevibacter*	**↑**	**–**	Wallen et al., 2022

↑ = increase, ↓ = decrease, – = no significant change. Microbial signatures were identified using 16S rRNA gene sequencing of fecal samples, with differential abundance determined using standard pipelines as reported in the original studies. PD, Parkinson’s disease; AD, Alzheimer’s disease.

*Firmicutes*, encompassing key SCFA producers such as *Faecalibacteriaceae* and *Lachnospiraceae*, have been consistently reduced across several studies on brain aging (Koutsokostas et al., 2024; Sun et al., 2019). In contrast, *Proteobacteria* and *Actinobacteria* were elevated, reflecting a pro-inflammatory microbial environment (Koutsokostas et al., 2024). At the genus level, reported increases in *Methanobrevibacter* and *Megasphaera* in PD suggest alterations in methane production and lactate metabolism, potentially affecting gut motility and immune regulation (Cabral and Weimer, 2024; Ghoshal et al., 2016). Similarly, multiple studies have reported higher levels of *Collinsella* and *Subdoligranulum* in PD (Zeng et al., 2024) and AD (Borrego-Ruiz and Borrego, 2025; Rob et al., 2025), linking these taxa to oxidative stress, dopaminergic neuron damage, and anxiety-related phenotypes (Dias et al., 2013; Mendez, 2021). Comparative analysis reveals a mix of overlapping and distinct microbial patterns across the different NDs. PD and AD share alterations in *Lactobacillus* (Chui et al., 2024), *Collinsella* (Rob et al., 2025), *Prevotella* (Chui et al., 2024), and *Akkermansia* (Heravi et al., 2023), pointing to overlapping mechanisms involving neuroinflammation, protein misfolding, and neurotransmitter dysregulation. Conversely, emerging literature reported a decrease in *Blautia* abundance in PD, therefore, presenting a significant limitation for drawing overarching conclusions. These microbial shifts reflect disrupted gut-brain communication characterized by systemic and CNS inflammation, impaired blood-brain barrier integrity, and altered metabolite production.

Collectively, many of these ND-associated microbial signatures mirror those observed in healthy aging populations, including reduced *Firmicutes*, enrichment of *Proteobacteria* and *Verrucomicrobia*, and loss of SCFA-producing taxa (Koutsokostas et al., 2024). These shared alterations suggest that age-related dysbiosis may lay the foundation for neuropathological changes, with disease states representing more advanced conditions.

### 3.2 Metabolite alterations

Gut microbiota-derived metabolites have been associated with neuronal function, influencing neuroinflammation, neurotransmission, and disease pathophysiology (Rob et al., 2025; Table 2).

**TABLE 2:** Microbiota metabolites in neurodegenerative diseases.

Disease	Altered metabolites in the colon	References
PD	**↑** Proline, **↑** Glutamate, **↓** Histidine, **↓** Nicotinamide, **↑** BCAA, **↓** SCFAs, **↑** Homocysteine, **↑** Folate	Luzzi et al., 2022; Rob et al., 2025; Zhang Y. et al., 2022
AD	**↑** Fructose, **↑** Mannose, **↑** Lysine, **↓** Aromatic AAs, **↑** Homocysteine, **↑** Folate, **↓** SCFAs	Fenech, 2017; Griffin and Bradshaw, 2017; Luzzi et al., 2022
Shared Across PD + AD	**↓** SCFAs, **↑** Homocysteine, **↑** Folate, **↓** Aromatic AAs	Rob et al., 2025
Common to PD and AD	**↑** Homocysteine, **↓** SCFAs	Rob et al., 2025

↑ = increase, ↓ = decrease. Metabolite profiles were derived from fecal or serum samples, depending on the study, using metabolomics as described in the original publications. PD, Parkinson’s disease; AD, Alzheimer’s disease; BCAA, branched-chain amino acids; SCFAs, short-chain fatty acids; Aromatic AAs, aromatic amino acids.

#### 3.2.1 Short-chain fatty acids (SCFAs)

Short-chain fatty acids such as acetate, propionate, and butyrate, primarily produced by *Firmicutes* and *Bacteroidetes*, maintain intestinal barrier integrity, regulate T-reg/Th17 balance, and modulate neuroinflammation (O’Riordan et al., 2022; Silva et al., 2020). Several studies have shown a positive correlation between SCFA-producing bacteria like *Faecalibacterium*, *Lachnospiraceae*, and *Veillonella*, with PD and AD. This deficiency impairs T-reg function, increases Th17-driven pro-inflammatory cytokines (IL-17A, TNF-α), and contributes to α-synuclein and amyloid-β aggregation, accelerating neurodegeneration (Yang et al., 2017; Zenaro et al., 2015). In addition, SCFAs bind to G-protein coupled receptors (GPR41, GPR43, FFAR2/3, GPR109A) expressed on immune and neural cells, initiating anti-inflammatory signaling cascades that stabilize the BBB and regulate microglial maturation (Choe, 2025; Loh et al., 2024). These receptors mediate direct modulation of microglial activation states, reduction of pro-inflammatory cytokine production, and enhancement of neurogenesis (Cheng et al., 2024; Silva et al., 2020). Acetate supports microglial development and phagocytosis, while butyrate acts as a histone deacetylase (HDAC) inhibitor, promoting gene expression patterns that protect against neurodegeneration (Erny et al., 2021). However, SCFA production tends to decline with aging-associated dysbiosis, potentially weakening these protective mechanisms. This decline was observed in preclinical studies, where decreases in butyrate levels were associated with cognitive decline (Chilton, 2024; Mishra et al., 2024).

The gut-brain connection involves a network of microbial metabolites and host pathways that influence brain physiology (Kasarello et al., 2023; Morais et al., 2021). Existing literature reports that dysregulated microbiota accelerates brain aging by increasing oxidative stress, mitochondrial dysfunction, and chronic neuroinflammation (Chaudhari et al., 2022; Clemente-Suárez et al., 2023b; Mattson and Arumugam, 2018; Picca et al., 2020). In AD, impaired mitochondrial regulation is associated with reduced expression of PGC1α, NRF2, and TFAM in postmortem brain tissues (De Plano et al., 2023). These mitochondrial regulators are influenced by microbiota-derived SCFAs. Butyrate acts as a histone deacetylase inhibitor, regulating gene expression related to mitochondrial biogenesis and antioxidant defense to directly impact brain aging (Saban Güler et al., 2025). PGC1α also regulates brain-derived neurotrophic factor (BDNF), a neuroprotective molecule that declines with Aβ accumulation (Bi et al., 2024). Enhancing PGC1α expression in animal models restores mitochondrial content and increases BDNF levels, suggesting the potential to reverse some AD-related pathologies (Bi et al., 2024; Helli et al., 2024). Neuroinflammation exacerbates mitochondrial dysfunction and cognitive decline (Jo et al., 2021). Altered expression of the TREM2 gene, a regulator of microglial cells, has been implicated in brain aging, and the modulation of microbiota-derived SCFAs could potentially influence this process (Colombo et al., 2021). Overexpression of TREM2 in the hippocampus protects against neuroinflammation and improves cognition in mouse models (Sanjay Shin et al., 2022). These pathways provide a strong rationale for exploring interventions that target alterations in the microbiota to preserve neuroimmune homeostasis and delay brain aging.

#### 3.2.2 Other metabolites

Lipopolysaccharide is primarily produced by gram-negative bacteria of certain strains of *Escherichia coli*, *Salmonella enterica*, *Pseudomonas aeruginosa*, and *Bacteroides fragilis* (Munford, 2008). The pathogenic impact of LPS is exacerbated by compromised gut barrier integrity and systemic translocation of LPS to the brain, which contributes to neuroinflammation and α-synuclein aggregation (Carloni and Rescigno, 2023). In PD, LPS may trigger oxidative stress and neuroinflammation through reactive oxygen species (ROS) and activation of stress-related signaling pathways (Deng et al., 2020; Perez-Pardo et al., 2019). These processes compromise hippocampal integrity, exacerbate motor dysfunction, and promote α-synuclein accumulation, highlighting the pathogenic relevance of LPS-mediated gut-brain signaling.

Metabolomic profiling reported disease-specific amino acid disturbances in NDs (Table 2). PD patients were reported to have elevated branched-chain amino acids (BCAAs) and proline, in addition to reduced histidine and aromatic amino acids (Rob et al., 2025; Zhang Y. et al., 2022), while AD has been reported to be associated with altered fructose, mannose, lysine, and aromatic amino acid profiles (Griffin and Bradshaw, 2017). These amino acid profiles are shaped by the activity of the gut microbiota (Table 2). Dysregulated amino acids can impede neurotransmitter synthesis (serotonin, catecholamines, GABA), exacerbate oxidative stress, and contribute to BBB dysfunction (Coppola et al., 2013; Crabtree et al., 2016; Holeèek, 2020). Additional metabolites, including nicotinamide, folate, and homocysteine, have also been implicated in brain aging across several studies (Fenech, 2017; Luzzi et al., 2022; Rob et al., 2025). Reduced nicotinamide in PD has been observed to correlate negatively with high *Akkermansia* abundance, which influences host metabolism and vitamin synthesis (Rob et al., 2025). This shift in *Akkermansia* abundance may contribute to diminished mitochondrial function and antioxidant capacity (Brakedal et al., 2022). Collectively, existing literature emphasizes the essential role of the gut-brain axis in the development and progression of brain aging. These findings further offer the opportunity to develop novel therapeutic strategies to target the gut microbial composition and its metabolite pathways to prevent the progression of brain aging.

## 4 Microbiota composition in African populations compared to western cohorts

### 4.1 Overview of profiling gut microbiota in rural African communities

Communities are categorized based on their degree of urbanization, dietary patterns and living habit (de Lanerolle-Dias et al., 2015; Zhao et al., 2023). For instance, rural communities are generally small cities compared to urban habitats with a more complex network. In terms of dietary habits, urban populations tend to eat more ultra-processed foods than those living in rural areas, who generally consume traditional staple grains and minimally processed plant-based foods (Li and Lu, 2021). Several cross-sectional studies have characterized the gut microbiota of rural African populations consuming traditional diets rich in unprocessed plant foods, resistant starches, and fibers. Across different countries, including South Africa, Malawi, Tanzania, Cameroon, Liberia, Zimbabwe, Burkina Faso, Nigeria, and Uganda, rural communities exhibit microbial profiles dominated by *Prevotella*, *Bifidobacterium*, *Faecalibacterium*, *Ruminococcus*, and other fiber-degrading taxa (Allali et al., 2018; Ayeni et al., 2018; De Filippo et al., 2010; Lokmer et al., 2020; Morton et al., 2015; Oduaran et al., 2020; Ordiz et al., 2015, 2020; Osakunor et al., 2020; Rosa et al., 2018; Rubel et al., 2020; Schnorr et al., 2014). These taxa are often accompanied by a higher prevalence of *Lachnospiraceae* and *Ruminococcaceae*, reflecting the fermentation of complex polysaccharides (Flint et al., 2012). Many of these communities exhibit greater overall microbial richness and diversity compared to urban or Western populations, with a taxonomic structure adapted to fiber fermentation and SCFA production (de la Cuesta-Zuluaga et al., 2018). These taxa may also be associated with frequent contact with the natural environment, such as soil and vegetation, which is postulated to enrich the gut microbiota composition (Frame et al., 2019). High interaction with the natural environment has remained a major characteristic of rural communities, giving rise to the term “rural microbiome” (Du et al., 2021). Various research demonstrates that the rural microbiome can enhance health and protect against chronic illnesses (Blaser, 2017; Zuo et al., 2018). In contrast, studies conducted in urban African settings have revealed more variable gut microbiota compositions, often characterized by a relative increase in *Clostridium* and *Bacteroides* taxa, along with a decline in the *Prevotella* genus (Ayeni et al., 2018; Rocafort et al., 2019; Samb-Ba et al., 2014; Smith et al., 2013). African cohorts with nutrient-deficient, metabolic, or infectious conditions (Allali et al., 2018; Doumatey et al., 2020; Fassatoui et al., 2019; Lokmer et al., 2020; Oduaran et al., 2020; Ordiz et al., 2015, 2020; Osakunor et al., 2020; Parbie et al., 2021; Rubel et al., 2020; Salah et al., 2019; Smith et al., 2013; Tang et al., 2017) also reported a decline in *Prevotella* taxa, indicating that urbanization, westernized diets, and disease burden may contribute to a microbiota transition toward less fiber-fermenting and more proteolytic bacterial taxa. This shift aligns with a pattern of reduced microbial diversity and potential pro-inflammatory signatures, commonly associated with metabolic disorders and compromised gut integrity (Severino et al., 2024). These findings highlight the emerging differences between the traditional, *Prevotella*-enriched “rural microbiome” and the increasingly heterogeneous, *Clostridium*/*Bacteroides*-dominated “urban microbiome,” shaped by modernization and changing nutritional environments. Therefore, longitudinal studies of cognitive function in rural African cohorts, in relation to microbiota profiles to quantify neuroprotective performance associated with their traditional diets, are necessary.

### 4.2 Gut microbial differences across different populations

Urbanization is associated with rapid dietary and lifestyle shifts, including increased consumption of refined carbohydrates, animal products, and processed foods (Casari et al., 2022). Comparative studies within Africa reveal clear microbial stratification between rural and urban populations. Rural residents typically maintain high *Prevotella*-to-*Bacteroides* ratios and harbor fiber-adapted communities. In contrast, urban populations show declining *Prevotella* abundance and a gradual shift toward microbiota configurations resembling Western profiles. Urban cohorts often present reduced diversity, lower abundance of SCFA producers, and increased representation of opportunistic or potentially inflammatory species of Bacteroides and Enterobacteriaceae (Abjani et al., 2023; Ecklu-Mensah et al., 2023; Huang et al., 2023). These trends mirror the ongoing nutrition transition and may reflect the impact of reduced dietary fiber and increased fat and sugar intake on microbial ecology.

Western populations have reported microbial communities that are less diverse and skewed toward taxa adapted to simple carbohydrates and animal-based substrates (Beam et al., 2021; Clemente-Suárez et al., 2023a; Makki et al., 2018). These microbiota profiles are characterized by higher *Bacteroides* prevalence, lower *Prevotella*, and reduced SCFA production capacity. Several studies have also shown a decrease in mucin-degrading capacity and thinning of the intestinal mucus layer, resulting in increased gut permeability and inflammation in Western populations (Desai et al., 2016; Makki et al., 2018; Suriano et al., 2022). Comparative analyses between African rural communities and Western cohorts consistently show marked differences in microbial structure and function, with African groups exhibiting higher levels of saccharolytic fermentation and enriched pathways for the degradation of complex carbohydrates (Brewster et al., 2019; De Filippo et al., 2010). These differences highlight observed disparities in inflammatory profiles and may contribute to differential trajectories of brain aging across populations (Shi et al., 2021; Zhang F. et al., 2022). In a comparative study using Malawian and Finnish children fed the same diets, but under different environmental conditions, significant changes in the microbiome and specific taxa distribution were observed. *Bifidobacteria* were dominant in all infants, with a greater proportion in Malawian (70.8%) than in Finnish infants (46.8%). Additional distinctions among bacterial genera were observed, with species like *Bifidobacterium adolescentis*, *Clostridium perfringens*, and *Staphylococcus aureus* being absent in Malawian but detected in Finnish infants (Grześkowiak et al., 2012). These differences suggest that African microbiota profiles may have greater protection against neuroinflammation through sustained SCFA production and mucosal integrity, but rapid urbanization threatens these benefits. In contrast, the Westernized gut microbiota, marked by lower microbial diversity (D’Aloisio et al., 2025) and reduced SCFA-producing taxa (Agus et al., 2016), reflects dietary shifts toward refined carbohydrates and animal-based fats. This profile is associated with heightened intestinal permeability (Severino et al., 2024), systemic inflammation (Christ et al., 2019), and decreased neuroprotective signaling (Kanoski and Davidson, 2011), all of which are risk factors for accelerated cognitive decline and neurodegenerative disease (Noble et al., 2017; Solch-Ottaiano et al., 2023).

## 5 Traditional African diets: regional differences, composition, processing methods, and cultural practices

Diet is arguably the most potent and consistent modulator of the human gut microbiota, capable of inducing significant shifts in its composition and function within days (David et al., 2014). The striking disparities in the metabolic output of the microbiome among people living in various regions with different cultures can mainly be ascribed to long-term dietary habits. This section explores the characteristics of traditional African diets, examines the specific ways in which their components shape the gut microbiome, and draws parallels with evidence from Western interventional studies that validate these diet-microbe interactions.

### 5.1 The composition and diversity of traditional African diets

It is a common oversimplification to refer to a single “African diet.” The continent is home to immense ecological and cultural diversity, resulting in a wide array of dietary patterns. However, many traditional, rural African diets share core characteristics that distinguish them from the typical Western diet, which is high in fat, sugar, and low in fiber (Statovci et al., 2017). Predominantly, these diets are plant-based and exceptionally rich in dietary fiber, derived from a variety of sources, including whole grains, legumes, tubers, fruits, and vegetables, all of which are rich in resistant starches and complex carbohydrates crucial for gut microbial diversity and function (Aworh, 2023). Those found in staples vary by region, but may include minimally processed cereals such as sorghum, millet, and teff, and starchy tubers like cassava and yams (Ghosh et al., 2023). Complex carbohydrates or resistant starches are high in these staples and cannot be digested in the upper portion of the gastrointestinal tract but are fermented in the colon. In rural South Africa, for example, many diets focus on maize meal porridge and legumes, and fiber consumption regularly exceeds 50 g per day, almost 3 times the daily intake in most Western nations, which typically range from 15 to 20 g per day (O’Keefe et al., 2015; Papier et al., 2025; Quagliani and Felt-Gunderson, 2017). In Burkina Faso, traditional diets consist of large amounts of sorghum, millet, and vegetables, hence, an abundance of fiber-degrading bacteria in their gut microbiome (De Filippo et al., 2010).

A typical characteristic of most traditional African food practices is heavy reliance on the fermentation process as a means of food preparation and preservation. This practice extends the shelf-life of foods and also introduces live microorganisms (probiotics) and their beneficial metabolites (postbiotics) (Marco et al., 2021). Natural fermented foods (for example, fufu, a fermented cassava meal in West African countries; ogi, a sour maize porridge in Nigeria; injera, a sourdough flatbread in Ethiopia made with teff; and mahewu, a non- alcoholic fermented maize beverage in Southern Africa) are part of the daily diet (Siddiqui et al., 2023). These foods are known sources of SCFA-producing bacteria, such as *Lactobacillus* and *Bifidobacterium* species (Malongane and Berejena, 2024; Mgbodile and Nwagu, 2023). This habitual use of fermented foods contrasts with the Western diet, where fermented products are less prevalent and the intake of microbes is restricted by sterilization and food processing.

### 5.2 Dietary patterns and the gut microbiota

The distinct composition of traditional African diets directly fosters a unique gut microbial ecosystem. The high availability of diverse, non-digestible carbohydrates is the primary driver behind the enrichment of specific bacterial taxa that are less abundant in populations consuming a Western diet. The most widely reported marker of a high-fiber diet of non-Western origin is a high proportion of the genus *Prevotella* (Gorvitovskaia et al., 2016). In addition to *Prevotella*, fiber-rich diets promote the growth of butyrate-producing bacteria, which are important for gut and systemic health. Abundances of *Faecalibacterium*, *Roseburia*, and *Ruminococcus* have been reported to be potent fermenters of resistant starch and dietary fiber, resulting in the formation of short-chain fatty acids (SCFAs), specifically butyrate (Koh et al., 2016). Butyrate is the primary energy source for colonocytes, thereby enhancing the integrity of the gut barrier. Additionally, traditional Africans’ diets are low in processed foods and artificial sweeteners, and high in saturated fats (Oniang’o et al., 2025). This usual intake is hypothesized to promote overall health by supporting the diversity of the gut microbiota (De Filippo et al., 2010), enhancing immune response (Temba et al., 2025), and influencing hormone secretion through microbial metabolites (Samami et al., 2025).

Although some of the most effective correlational studies are based on cross-sectional research in African populations, dietary intervention studies, usually conducted in Western cohorts, provide causal evidence of the rapid and drastic effect of diet on the microbiome (Wilson et al., 2020). One of the most compelling pieces of evidence comes from the dietary swap study by O’Keefe et al. (2015). In this pioneering research, rural Africans were placed on a low-fiber, high-fat Western diet, while African Americans consumed a high-fiber, low-fat traditional African diet. Participants who switched to the African-style diet showed a marked increase in saccharolytic fermentation and butyrate production, accompanied by reductions in colonic mucosal markers of inflammation and proliferation, both of which are considered biomarkers of colorectal cancer risk. In contrast, those on the Western-style diet exhibited changes associated with a higher risk of disease. Furthermore, treatments that administer certain types of fiber have also reported measurable shifts in the bowel microbiome. Supplementation with inulin-type fructans, galactooligosaccharides (GOS), and inulin has been shown to specifically increase the abundance of *Bifidobacterium* species (Carlson et al., 2018). This evidence suggests that a diet dominated by complex plant polysaccharides is a consistent choice that can support a healthy and metabolically resilient microbiome. The eating habits of traditional rural African societies, marked by increased consumption of fiber and fermented foods, are essential for maintaining a healthy gut, thereby promoting healthy brain aging.

### 5.3 Dietary patterns, gut microbiota, and brain aging in African populations

Longitudinal studies linking gut microbiota profiles to brain aging and neurodegenerative disease outcomes in African populations are limited. However, epidemiological and nutritional evidence support the biological mechanisms discussed in this review. According to the Institute of Health Metrics and Evaluation (IHME), African regions have an Alzheimer’s disease and other dementia prevalence of 0.2% as of 2021, while South America, North America, Europe, and Eastern Asia and Pacific had a reported prevalence of 0.7%, 1.5%, 1.6%, and 1.1%, respectively (IHME and Global Burden of Disease, 2024). Limited surveillance infrastructure in Africa has contributed to uncertainty in these estimates. However, recent studies indicate a rapid rise in age-related neurodegenerative disorders as populations age and urbanization accelerates across the continent (Olajide et al., 2025).

**FIGURE 2:**
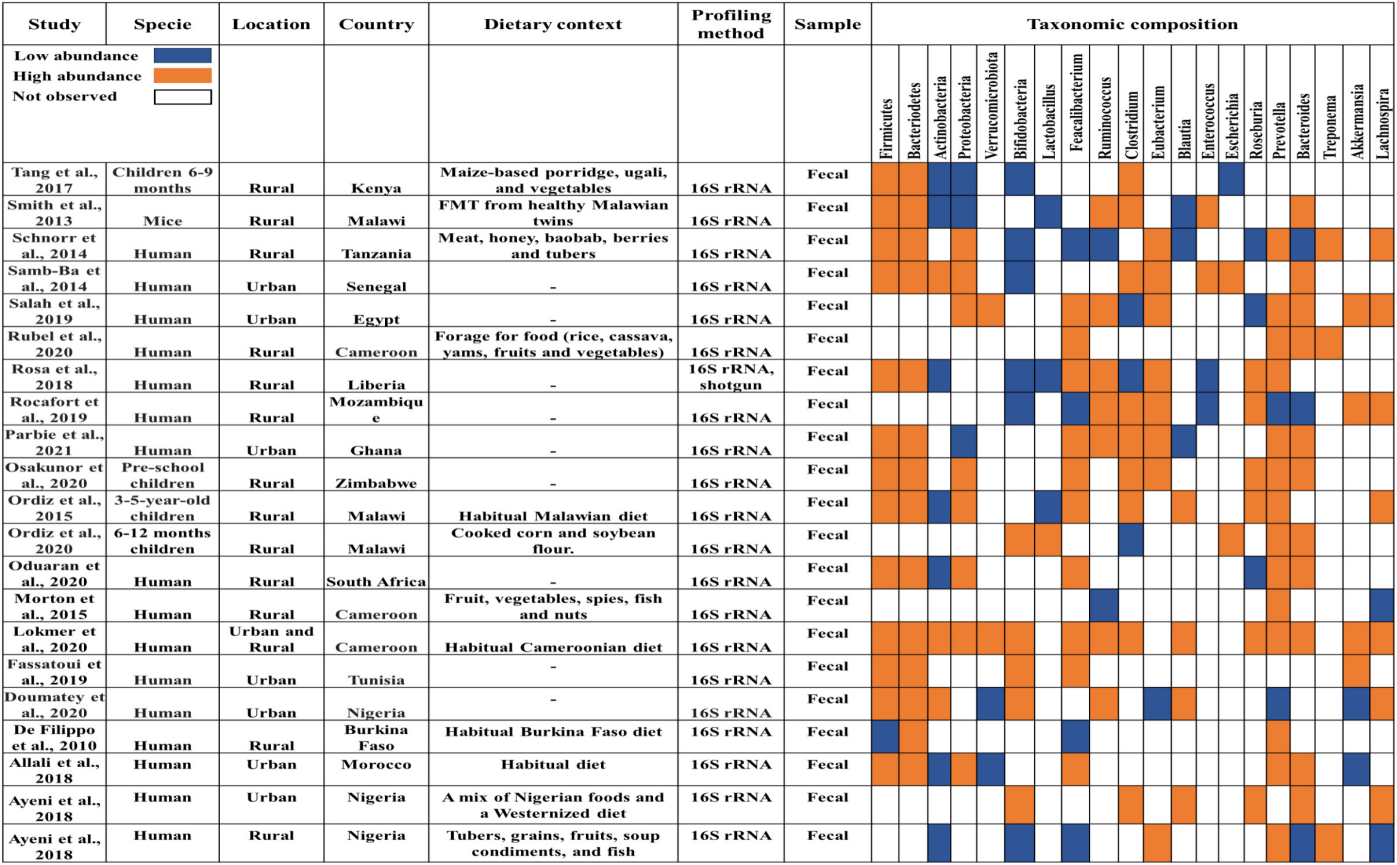
Summary of the gut microbiota compositions in African communities. We summarize gut microbiota composition reported across African populations, including both rural and urban cohorts. The review encompasses African populations broadly. However, rural communities consuming traditional, fiber-rich diets are emphasized due to their distinct and consistently reported microbiota signatures, particularly Prevotella-enriched and SCFA-producing profiles. This comparative framing highlights how dietary patterns shape gut microbiota composition across Africa. FMT, Fecal Microbial Transplantation.

This emerging epidemiological transition coexists with heightened dietary shifts. Traditional African diets are increasingly being replaced in urban and peri-urban settings by Westernized dietary patterns (Ameye et al., 2025). These dietary changes align with alterations in gut microbiota composition observed in urban African populations (Figure 2). Additionally, the microbial composition enriched in traditional rural African diets aligns closely with mechanisms that support brain health (Isibor et al., 2021). High SCFA production, enhanced gut barrier integrity, and reduced systemic inflammation have been associated with neurogenesis, reduced microglial activation, improved mitochondrial health, and attenuation of neuroinflammatory signaling (Jabbari Shiadeh et al., 2025). In contrast, microbiota alterations associated with Westernized diets are consistently linked to increased gut permeability, chronic low-grade inflammation, and impaired neuroprotective signaling pathways. All of which are recognized contributors to accelerated brain aging and neurodegeneration (Wiêckowska-Gacek et al., 2021). These population-level and mechanistic observations support a biological framework that the traditional African diet may influence brain aging through microbiota-mediated pathways.

## 6 Proposed conceptual framework: traditional African diet, gut microbiota, and brain aging

**FIGURE 3:**
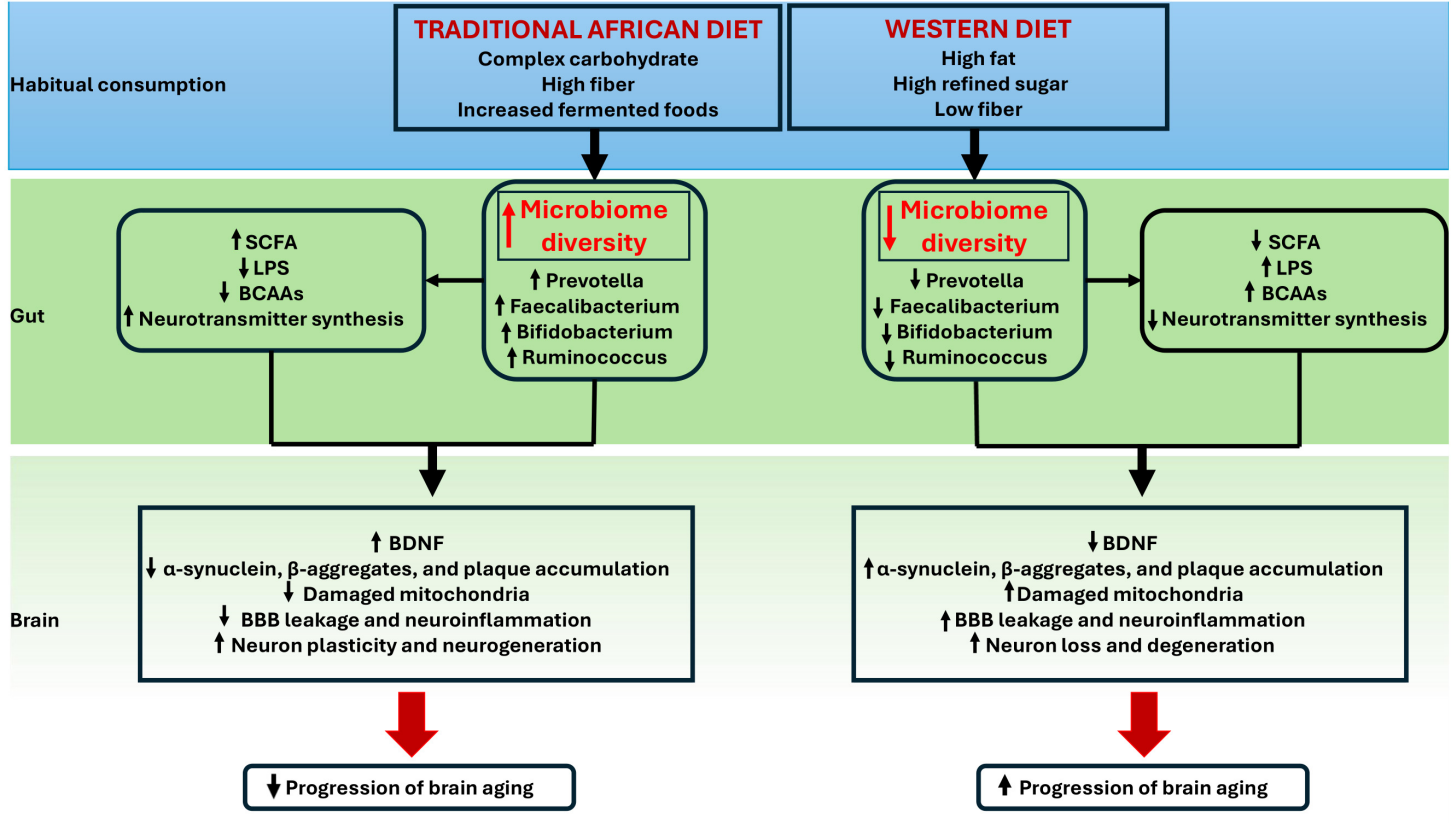
Proposed framework linking traditional African dietary patterns, gut microbiota composition, and brain aging. Traditional African diets enriched with fiber and fermented foods support beneficial microbial communities, which in turn may promote neuroprotective processes. In contrast, Western diets may disrupt these interactions, accelerating neurodegeneration and brain aging. Data supporting associations between traditional African diets, gut microbial diversity, and enrichment of taxa are primarily derived from cross-sectional studies in African populations (Ayeni et al., 2018; Ordiz et al., 2015, 2020). In contrast, mechanistic links connecting microbial metabolites, neuroinflammation, mitochondrial function, and cognitive outcomes are largely informed by animal models and human studies conducted in Western populations (Kanoski and Davidson, 2011; Severino et al., 2024; Shi et al., 2021). As such, several pathways in this framework are biologically plausible hypotheses rather than confirmed causal relationships within African cohorts. SCFAs, short-chain fatty acids; LPS, lipopolysaccharide; BCAAs, branched-chain amino acids; BDNF, brain-derived neurotrophic factor.

Synthesizing the evidence presented, we propose a conceptual framework that highlights how traditional African dietary habits may shape gut-brain interactions relevant to brain aging (Figure 3). Each component of the framework is supported by evidence discussed in Sections “3 The role of gut microbiota in brain aging–5 Traditional African diets: regional differences, composition, processing methods, and cultural practices,” with microbial signatures and metabolites outlined in Section 3–5. Traditional African diets, comprising complex carbohydrates, high fiber, and frequent consumption of fermented foods, are associated with higher microbial diversity and enrichment of SCFA-producing taxa, including *Prevotella*, *Faecalibacterium*, *Bifidobacterium*, and *Ruminococcus*. These taxa promote SCFA production, reduce lipopolysaccharide exposure, and improve neurotransmitter synthesis. These processes may support higher BDNF levels, reduced neuroinflammation, and enhanced neuronal plasticity, thereby delaying brain aging. In contrast, Western dietary patterns, typically low in fiber and high in refined sugars and fats, may disrupt these interactions, leading to reduced microbial diversity and accelerated neurodegenerative processes. This framework synthesizes existing knowledge and proposes novel hypotheses regarding the differential neuroprotective potential of traditional African diets. Furthermore, this framework serves as a predictive model for understanding how urbanization and the westernization of diets in Africa might accelerate brain aging by disrupting beneficial microbiota-gut-brain interactions, specifically by predicting shifts in *Prevotella*-to-*Bacteroides* ratios to identify novel intervention targets specific to populations undergoing transitions. This framework provides a foundation for hypothesis-driven research in African populations. Future studies could test these pathways using longitudinal cohort designs integrating dietary assessments, gut microbiome profiling, circulating inflammatory and metabolic biomarkers, neurotrophic factors such as BDNF, and standardized cognitive outcomes. Dietary intervention studies examining rural-to-urban transitions are important for evaluating microbiota-mediated mechanisms of brain aging in African populations.

## 7 Research gaps and future directions

While conversations and emerging evidence on the human microbiome, including its connection to brain aging, are growing globally, this review has shown that the African population is substantially underrepresented in available data. Furthermore, the current limited evidence remains largely concentrated in a few countries and primarily targets indigenous or rural populations. This limited geographic and demographic representation affects the generalizability of findings across the continent, thus demonstrating the need for more concerted evidence generation to address this critical research gap. Dietary influences on shaping the gut microbiome have been established in high-income settings, with concrete evidence from studies on Mediterranean diets (Barber et al., 2023; De Filippis et al., 2016; Perrone and D’Angelo, 2025). Specific fiber interventions with evidence supporting dietary modulation and prebiotic interventions on gut health and brain function have also been investigated (Becker et al., 2022). In contrast, pooled evidence suggests that studies from Africa are cross-sectional and descriptive in design, limiting the ability to draw causal inferences about diet-microbiome-brain interactions. There is a need to invest in longitudinal and interventional studies that incorporate the effect of regions’ diverse dietary patterns, cultural contexts, and environmental exposures in these interactions. This will deepen unique region-specific insights rather than extrapolate evidence from populations with different genetic and environmental backgrounds. In a period marked by global funding constraints and widening health disparities, it is crucial to recognize that populations in resource-limited settings, often characterized by restricted access to quality healthcare and lower overall living standards, face a disproportionately higher risk of adverse brain aging outcomes. Therefore, a shared commitment to mobilizing resources and strengthening research capacity across Africa is vital. This includes establishing dedicated funding streams specifically for African-led, collaborative gut microbiome and neurocognitive research, prioritizing indigenous research questions and methodologies. Furthermore, promoting collaborative training programs for African scientists in advanced techniques for gut microbiome analysis, neurocognitive assessment, and biostatistics is essential, alongside ensuring all research is conducted within robust ethical frameworks. Future research should also employ longitudinal and intervention studies, such as randomized, community-based dietary intervention studies comparing traditional high-fiber diets against Westernized dietary patterns in age-matched African cohorts, utilizing multi-omics profiling, neurocognitive assessments, and systemic inflammatory markers. This would provide novel causal insights into the diet-microbiome-brain axis, overcoming the limitations of cross-sectional designs. To enhance reproducibility, future studies must prioritize comprehensive disclosure of all experimental parameters, precise bioinformatics pipelines, sequencing platforms, and detailed specifications of analytical equipment used for metabolomics and neuroimaging.

## 8 Conclusion

The contribution of gut microbiota interactions with the brain in mitigating brain aging and reducing the risk of neurodegenerative disease has gained scholarly attention. However, African populations remain underrepresented in this growing body of evidence. To bridge this gap and identify critical research needs, we synthesized existing literature linking dietary patterns, microbial composition, and metabolite profiles to brain aging in Africa compared with Western counterparts. Findings indicate that favorable microbial profiles, particularly those enriched with specific *Prevotella* species, may be associated with traditional African diets characterized by high fiber intake and frequent consumption of fermented foods. These dietary patterns promote the abundance of short-chain fatty acid (SCFA)-producing taxa such as *Faecalibacterium* and *Roseburia*, which are linked to improved gut and brain health. In contrast, evidence from diet-related clinical and interventional studies on Alzheimer’s and Parkinson’s diseases in high-income settings shows that Western dietary patterns, typically dominated by *Bacteroides* and other pro-inflammatory taxa, are associated with reduced SCFA production, elevated lipopolysaccharide exposure, and disruptions in amino acid and nicotinamide metabolism. Thus, these comparisons have shown that diet is integral in modulating gut microbiota composition and metabolic outputs, ultimately affecting inflammation and brain aging.
