# Supplementary figures and images for: Gut microbiota and brain aging: a comparative review of African and western populations
Source: Front Aging Neurosci. 2026 Feb 12;18:1740408. doi: 10.3389/fnagi.2026.1740408 (PMC12935954; doi:10.3389/fnagi.2026.1740408)

## *Supplementary Material*

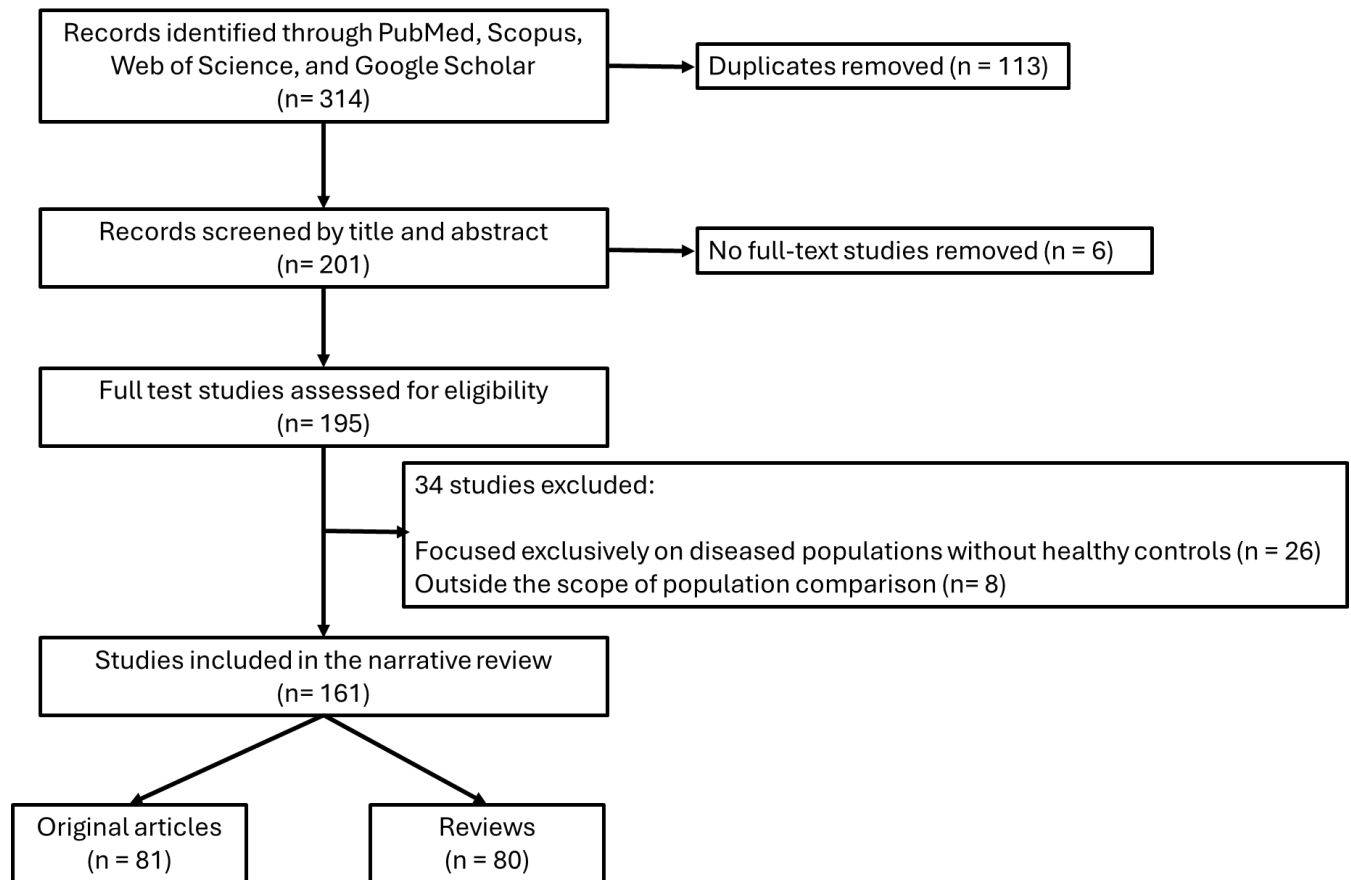

**Supplementary Figure 1. Flow diagram of narrative review**

Supplement: Supplementary file 1 [file Data_Sheet_1.pdf]
